# Computerized spatial delayed recognition span task: a specific tool to assess visuospatial working memory

**DOI:** 10.3389/fnagi.2015.00053

**Published:** 2015-04-24

**Authors:** Corina Satler, Flávia Schechtman Belham, Ana Garcia, Carlos Tomaz, Maria Clotilde H. Tavares

**Affiliations:** ^1^Faculty of Ceilandia, University of BrasiliaBrasilia, Brazil; ^2^Institute of Cognitive Neuroscience, University College LondonLondon, UK; ^3^Laboratory of Neurosciences and Behavior, Department of Physiological Sciences, University of BrasiliaBrasilia, Brazil; ^4^CEUMA University/UniCEUMASão Luís, Brazil

**Keywords:** visuospatial abilities, working memory, tablet device, psychology software tools, aging, non-human primates, neuropsychological tests, electrophysiology

## Abstract

A new tablet device version (IOS platform) of the Spatial Delayed Recognition Span Task (SDRST) was developed with the aim of investigating visuospatial Working Memory (WM) abilities based on touchscreen technology. This new WM testing application will be available to download for free in Apple Store app (“SDRST app”). In order to verify the feasibility of this computer-based task, we conducted three experiments with different manipulations and groups of participants. We were interested in investigating if (1) the SDRST is sensitive enough to tap into cognitive differences brought by aging and dementia; (2) different experimental manipulations work successfully; (3) cortical brain activations seen in other WM tasks are also demonstrated here; and (4) non-human primates are able to answer the task. Performance (scores and response time) was better for young than older adults and higher for the latter when compared to Alzheimer’s disease (AD) patients. All groups performed better with facial stimuli than with images of scenes and with emotional than with neutral stimuli. Electrophysiology data showed activation on prefrontal and frontal areas of scalp, theta band activity on the midline area, and gamma activity in left temporal area. There are all scalp regions known to be related to attention and WM. Besides those data, our sample of adult captive capuchin monkeys (*Sapajus libidinosus*) answered the task above chance level. Taken together, these results corroborate the reliability of this new computer-based SDRST as a measure of visuospatial WM in clinical and non-clinical populations as well as in non-human primates. Its tablet app allows the task to be administered in a wide range of settings, including hospitals, homes, schools, laboratories, universities, and research institutions.

## Introduction

Memory involves the ability to acquire, retain and utilize information and knowledge. This fundamental process allows learning and adaptive behavior, considering that the organism can use its previous experiences to select the most appropriate behavior for the upcoming situation (Simon and Kaplan, 1990; La Cerra and Bingham, 1998). Particularly, the term Working Memory (WM) is used in reference to a broad framework of interacting processes that involve the temporary storage and manipulation of information in the service of performing complex cognitive activities (Baddeley et al., 2011).

The conception of WM grew up of the literature on short-term memory (STM) in the mid 1970s (Baddeley and Hitch, 1974) and soon became the most influential empirical model of cognitive functions (Baars and Franklin, 2003). There is a wide consensus in the literature describing the processes that underlie WM, including (a) active maintenance of ordered information for relative short periods of time; (b) context-relevant updating of information, and goal-relevant computations involving active representations; (c) rapid control of task-relevant cognitions and behaviors in the service of currently pursued goals (O’reilly et al., 1999; Gazzaley, 2011). These functions are relevant for a successful cognitive interaction between intern and extern stimuli and make WM a central topic of research in the field of general psychology. Additionally, WM has different aspects, such as capacity (load), time (decay of speed), and control of attention. Regarding the limits of WM, studies have shown that several animals, including humans, are able to visually scan an entire field when looking for predators or prey. However, only a small part of information is retained for detailed analysis (Cowan et al., 2008).

In 1887, Joseph Jacobs, researching on STM, introduced the so-called digit span to measure the capacity of STM. The task consisted of presentation of a random series of digits which participants had to repeat in their correct serial order. The longest sequence correctly repeated was comprized by approximately 6 or 7 items. That figure was understood as the capacity of STM. This limited capacity to remembering some information (named “chunk capacity limits”), was later reported by Miller (1956) in his classical article “The magic number seven”. He defined the number seven (given or taken two) as a limit of our capacities to hold at once some information in immediate memory. Afterwards this memory capacity was also associated with WM due to its mechanism to maintain in mind goals and other information needed to complete a task (Miller et al., 1960).

In a more recent development of his model, Baddeley describes four main components of WM: a control system of limited attentional capacity called the central executive; the episodic buffer, responsible for the association of new and old information; and two subsidiary storage systems, named the phonological loop—holds and manipulates speech-based information, and the visuospatial sketchpad (VSSP) performs a similar function for visual and spatial information (Baddeley, 2003; Baddeley et al., 2011). According to this model, the VSSP is related to representations of visual appearance that are organized at the level of objects and is helpful for locating them in relation to visual perceptual analysis (Baddeley and Hitch, 1994).

In neuropsychology, there is abundant evidence linking the dorsolateral areas of the prefrontal cortex (DLPFC) to a range of executive processes, including the processing and maintenance of spatial stimuli in WM (Rudkin et al., 2007). In this line, Zarahn et al. (2000) conducted a functional magnetic resonance imaging (fMRI) study and found that the DLPFC were active during the maintenance of the relative location of sequentially presented spatial stimuli. Other fMRI study conducted by Leung et al. (2002) described activation of the DLPFC when participants were asked to retain sequentially presented spatial stimuli in a task involving high memory load. There is also evidence that posterior parietal and occipital cortices are involved in visuospatial WM (Jonides et al., 1993; Smith and Jonides, 1998). Premotor and right superior parietal cortices seem to mediate spatial storage and rehearsal, whereas inferior parietal areas mediate object storage (Smith and Jonides, 1998; Wager and Smith, 2003).

In most studies, WM is accessed using different types of neuropsychological tests, including standard pencil-and-paper tests and computerized tasks with different methods and apparatus. Particularly the concept of specialized visuospatial component has received a growing amount of attention over the last decades (Logie, 1995; Owen et al., 1998; Miyake et al., 2001; Rowe et al., 2008; Fiore et al., 2012) and numerous cognitive tasks have been proposed to investigate it.

Until today, span measures remain the gold standard for estimating WM capacity (Conway et al., 2005). In the Brooks matrix task, for example, a spatial sequence of locations within a matrix has to be memorized in correct order. In this task, it is assumed that the mental image of the locations is stored in VSSP. The spatial span test, also called the Corsi Block task (Milner, 1971) is structurally very similar. The task involves the recall of a sequence of movements. Participants have to memorize the items in the sequence in which they appear. That is, the items are only distinguishable by their spatial location.

The distinction between visual information (appearance) and spatial information (where the object are located) is a critical dimension in visuospatial WM. In this line, numerous modified versions of the spatial span task have been described in the attempt to develop a more sensitive paradigm for measuring visuospatial WM. However, one of the limitations of those tasks is the difficulty in applying them to general or clinical populations, such as older adults and Alzheimer’s disease (AD) patients, or in making a bedside evaluation. They are also difficult to being equally used by humans and non-human primates.

The “Spatial Delayed Recognition Span Task” (SDRST) have been used in an attempt to assess memory function within spatial and nonspatial domains. It has also been used to test memory capacity in aging or following a variety of neurologic disorders in non-human primates and humans. In general, the task requires the ability to identify a novel stimulus among an increasing array of previously presented stimuli using either spatial or nonspatial cues. Because participants have to hold spatial locations “online” and update them constantly to adjust to new information while answering the task, the SDRST is indeed tapping into visuospatial WM abilities (Lacreuse et al., 2005).

Therefore, using that paradigm as reference, we devised a new computer-based task to evaluate visuospatial WM abilities, specifically WM load. This cognitive tool can be run on a tablet device (IOS platform) and would be sensitive to cognitive differences brought by aging and neuropsychiatric disorders. It can also be used in non-human primate studies.

The use of mobile devices, such as tablets, for this type of testing has several benefits. First, the test is run on a tablet computer with touch screen technology. This feature could be very promising especially for older adults, considering that many of them are less familiar with computers. Second, computerized tests include better standardization in administration, precise stimulus control and scoring without manual operation. They record performance and reaction time accurately and can generate a mass of seemingly precise data developing large and accurate databases. Third, this new version of the task also permits the manipulation of experimental parameters such as presentation time, shape, type and other characteristics of stimuli, which generates numerous alternative forms suitable for repeated testing. Fourth, the SDRST application is available for free in Apple Store app (“SDRST app”), so the test can be taken at no-cost. Finally, it is important to note that the use of computerized cognitive tasks on portable devices allows assessments to be made in a wide range of environments. This task can be used, for instance, in the context of everyday life, schools, hospitals, laboratories, universities, and research institutions. Practitioners may also carry out tests when visiting patients.

In the current paper we discuss the format of the new task and its advantages by presenting data that evidences its successful use with different populations and types of stimuli.

## Materials and Methods

### Overview of the Spatial-Delayed Recognition Span Task (SDRST)

This test was developed by our group and runs in Delphi programming language for desktop using the computational program SYSMEN. It was presented in a touch screen monitor (LG Studio Works 440, Microtouch 17’) within arm’s reach distance. Additionally, this module was adapted in Objective C language, compatible with mobile devices on Apple/iOS platform.

**Figure 1:**
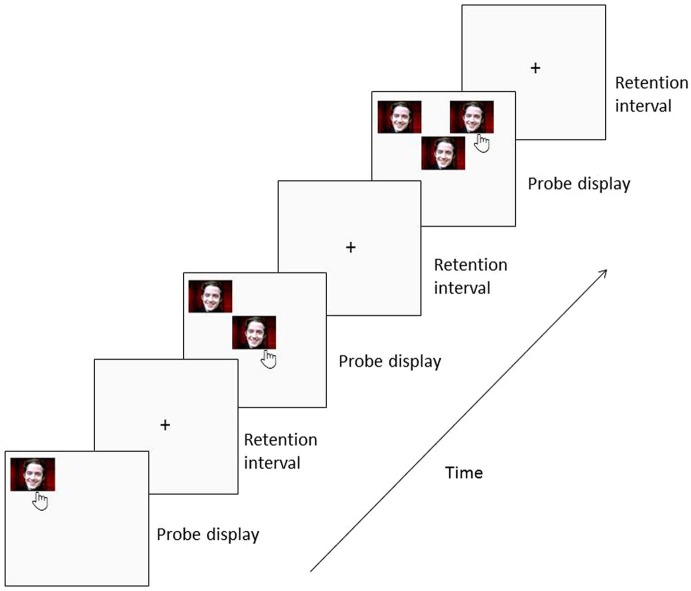
**Trial design for Unique condition**. Each trial began with a visual stimulus presented in one of the possible locations on the screen. Subjects have to touch it and it will disappear and reappear at the same location along with a second stimulus in a different location. These steps continue for the maximum number of stimuli pre-selected by the experimenter or until a mistake is made.

In this task, participants are required to discriminate a novel location of a stimulus among an increasing array of stimuli presented sequentially in various locations on the screen. A stimulus is presented in one of the possible locations on the screen and the participant has to touch it and reappears at the same location along with a second stimulus in a different location. The participant has to touch the stimulus that is in this new location, which will make both disappear and reappear at the same locations along with a third stimulus in a new location. These steps continue for the maximum number of stimuli pre-selected by the experimenter or until a mistake is made (Figure 1).

**Figure 2:**
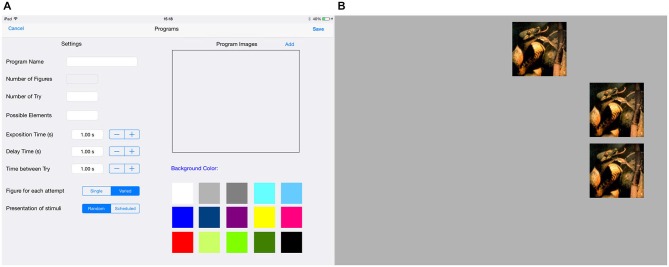
**(A)** Screen design shows all possible configurations for the stimulus. We can select the type of stimulus (Program stimuli); the background color (Screen color), the number of items and trials, possible elements, the exposition time, delay and interval time. Additionally, we can determine whether will be used the same stimulus repeatedly on the same trial or selected different stimuli (Stimuli for condition: Unique or Varied), and also can be defined if we want or not to display a randomly stimulus from the list (Stimuli choose: Random or Defined); **(B)** Example of trial design for Unique condition.

It is worth mentioning that the tool can be used in a wide variety of settings. The examiner can select the best configurations for the study: stimulus characteristics (colors, shapes, emotional valence, contextual images, faces, etc.), the size of the stimuli, the background color, the time of exposure of the stimuli on the screen, how many different stimuli will be used on the same trial and in the total of trials, how many trials and which stimuli will be used, how many stimuli will compose one trial, the interval time between two stimuli appearance, the distance between the screen and subjects, amongst others. Additionally, the examiner can determine whether the same stimulus will be shown repeatedly on the same trial or select different stimuli (Figure 2). Besides that, there is an option of giving participants an auditory feedback after each correct or wrong answer.

For research studies we suggest, when using the tablet, the device to be placed over a table or other hard surface that raises the height of the tablet. This will reduce motion artefacts, such as head movements and also remove muscle stress from the body. Nevertheless, one of the useful features of the SDRST is the flexibility with which test parameters can be chosen. Thus, we consider that examiners may place the tablet in the most appropriate way for their studies, taking into account the type and size of stimuli, as well as their objectives.

**Figure 3:**
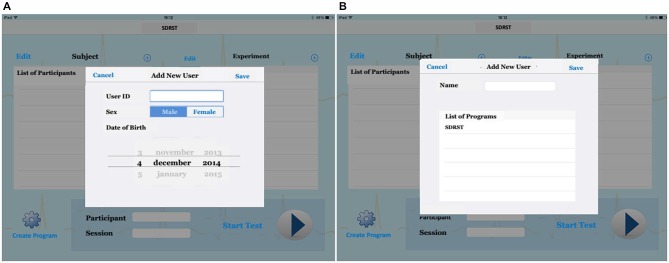
**(A)** We can register demographic information for new users, such as age, sex; After that, **(B)** we can choose the test that will be presented to the participant (List of Program: SDRST). Then, we can run our test.

The computer software registers demographic information of the participants (Figure 3), and correct and incorrect answers as well as the response time.

### Participants and Experimental Design

#### Participants

Three sets of studies were conducted using the SDRST, with a total of 172 participants. All participants were right-handed according to the Edinburgh Inventory (Oldfield, 1971). We give a summary of the participants and stimuli used on these studies now and present specific analysis and discussion on the next sessions.

Study “a” (Satler and Tomaz, 2011) included 22 AD patients (7 male, mean age 78.27 ± 6.70 years old) and 40 healthy older adults (16 males, mean age 71.10 ± 6.52 years old).

Study “b” (Belham et al., 2013) tested 27 young adults (13 males, mean age 21.26 ± 2.03 years old) and 25 older adults (14 males, mean age 69.72 ± 6.35 years old).

Study “c” (Garcia, 2011) tested 58 young adults (30 males; mean age 21.31 ± 2.77 years old).

Demographic details are shown in Table 1. All participants were right-handed, had normal or corrected-to-normal vision and hearing, reported not making g concomitant use of psychotropic medication, and were naive about the aims of the studies. A written informed consent in accordance with the ethical guidelines for research with human subjects (196/96 CNS/MS resolution) was obtained from all participants and their caregivers (where appropriate). The studies were approved by the Human Subject Committee of the Faculty of Medicine, and the Human Subjects Ethics Committee of the Health Sciences Faculty of the University of Brasilia.

**Table 1:** **Demographic characteristics of the groups**.

Characteristic	YA	OA	AD
N	84	64	22
Age, years	21.23 (2.63)	70.45 (6.55)	78.27 (6.70)
Sex (M/F)	43/41	30/34	7/15
Education, years	13.44 (1.49)	13.59 (5.12)	6.73 (4.00)

*Note. YA = young adults; OA = older adults; AD = Alzheimer’s disease patients. M/F = male to female. Values are mean (SD). Years of education of the age groups is statistically the same (t = −0.240; p = 0.811)*.

Both YA and OA were screened for cognitive impairment with the Mini-Mental State Examination—MMSE (Folstein et al., 1975) and AD patients met the criteria of AD described in the DSM-IV (1994) by the American Psychiatric Association and by the NINCDS-ADRDA (McKhann et al., 1984). These criteria were used as inclusion/exclusion criteria.

#### Experimental Design

Participants had to discriminate a novel location of a stimulus among an increasing array of stimuli presented sequentially in various locations on the screen. A stimulus was presented in one of the 16 possible locations on the screen and the participant had to touch it and the reappears at the same location along with a second stimulus in a different location. The number of stimuli would increase up to a maximum of 8 stimuli within one trial. All the stimuli were colored images sized 4 cm × 4 cm.

Study “a” and “c” used geometric pictures and IAPS images (Lang et al., 2008) with different emotional valences in two different task conditions. Participants performed 16 trials with each emotional valence in each task condition. The interval time between stimuli was 5 s—study “a” and 1 s—study “c”.

Study “b” used geometrical images and facial photographs representing negative, positive and neutral expressions. Each participant completed 10 trials with each emotional valence. The interval time between stimuli was 3 s. Correct answers led to an acute auditory feedback signal, and wrong answers, to a bass auditory signal.

Participants were received in the experimental room, where they read and signed the written informed consent. After that, they were invited to sit comfortably in front of the touch screen, which was positioned within the reach of the volunteer, and to answer the neuropsychological tests. Then, the instructions for the SDRST were read and participants answered one training session to verify if the test rules had been understood. Instructions were kept constant for all subjects. The time of execution of the task varied according to each participant’s response time, but the full procedure did not last more than 2 h. All participants used their dominant hand to perform the task.

## Results and Discussion

Several analyses were conducted in order to evaluate the feasibility of SDRST to tap into WM in different experimental conditions and with different groups of participants. Performance on the task was measured by the mean of corrected responses before a mistake in each trial. On the next sessions, we provide a brief description of the performance of participants (YA, OA and AD patients) using geometric, complex figures (International Affective Pictures System—IAPS, Lang et al., 2008) or facial stimuli of different emotional valences in two possible task conditions Table 2.

**Table 2:** **Performance on the SDRST for three groups of participants in different conditions**.

	Types of stimuli				Task condition				Emotional valence^a^					
	IAPS		Faces		Unique		Varied		Negative		Positive		Neutral	
	M	SD	M	SD	M	SD	M	SD	M	SD	M	SD	M	SD
YA	7.11	0.75	7.19	0.46	7.15	0.67	7.36	0.99	7.56	0.44	7.27	0.71	7.42	0.70
OA	4.96	1.57	5.96	0.94	5.35	1.42	6.41	1.34	6.16	1.06	5.85	1.02	5.90	1.22
AD	-		-		4.09	1.56	4.62	1.80	-		-		-	

*Note. YA = young adults; OA = older adults; AD = Alzheimer’s disease patients. ^a^ Emotional valence for facial stimuli*.

### Young, Older Adults and Alzheimer’s Disease Patients

Study “b” utilized geometrical pictures and emotional facial expressions as stimuli in a total of 40 trials (Belham et al., 2013) to investigate age-related differences. As expected, YA had a superior overall performance than OA (*F*_1,49_ = 42.787, *p* < 0.001), indicating that this task is a sensitive measure of the cognitive deficits brought by healthy aging in WM abilities.

Study “a” examined the WM performance in mild AD patients and healthy elderly controls, assessed with the SDRST (Satler and Tomaz, 2011) using IAPS images and geometrical pictures in a total of 16 trials. A mixed-design ANOVA revealed a major significant effect of group (*F*_1,60_ = 46.655, *p* < 0.001). The results showed that AD patients had more marked difficulties in performing the SDRST than OA, which were found to be related to difficulties in holding information in WM. This finding is congruent with a number of previous studies showing that AD is characterized by memory loss and cognitive impairment. Memory loss involves not only difficulties in learning new information but also in holding relevant information in mind over short periods of time (Kensinger et al., 2003; Huntley and Howard, 2010).

### Unique and Varied Conditions

On these analyses, we tested how participants’ performance would differ if we presented the task in distinct conditions: unique and varied. In the first one, all stimuli presented in the same trial were identical and only differ from trial to trial, whereas in the varied condition, stimuli were always different.

For these analyses, data of studies “a” and “c” were used. Analysis of Variance (ANOVA) showed that there was a main effect of the condition for YA (*F*_1,49_ = 333.96, *p* < 0.001), with the varied condition leading to a better performance than the unique condition. Condition also had a main effect for OA and DA (*F*_1,60_ = 14.92, *p* < 0.001; no interaction between groups and conditions, *F*_1,60_ = 0.374, *p* = 0.543), with the varied condition leading to a higher performance (Satler and Tomaz, 2011).

These results indicate that, when shown different stimuli on the same trial, participants have an extra mnemonic element to help responding the task. The variation on the content of the stimuli leads to a more active participation of the episodic buffer besides the regular visuospatial sketchpad activation. It also suggests that this happens regardless of the age or the psychological condition of the participant.

### Faces and IAPS Pictures

For this analysis, we were interested in investigating what would happen with young and older adults’ performance if we used two different types of stimuli: faces and scenes. Data from the three studies were analyzed. The faces were chosen based on a pilot study done with young and older adults and depicted adult models manipulated to only show the face with no interference from hair or other body parts. The scenes were selected from the IAPS, which is a set of static images containing various pictures depicting snakes, mutilations, accidents, illness, puppies, babies, and landscapes, among others (Lang et al., 2008).

Results indicate that there was a main effect of the type of stimuli (*F*_1,144_ = 7.979, *p* = 0.005), with faces leading to a better performance, though there was a significant interaction between age and type of stimuli (*F*_1,144_ = 6.361, *p* = 0.013). This data indicate that facial stimuli somehow facilitate information processing during the SDRST. This can be due to their biological importance (Palermo and Rhodes, 2007), small complexity when compared to scenes (Knight et al., 2007; Britton et al., 2006) or quicker detection (Liu et al., 2002).

### Negative, Positive and Neutral Emotional Valences

We used data from study “b” to investigate the influence of different emotional valences into WM during the SDRST. The facial images depicted anger, happiness or neutral expressions.

Data showed that negative images elicited a higher performance than positive images (*p* < 0.005) for both age groups tested, indicating that the modulatory effect of emotion on WM, and possibly the well established Negativity Bias (Baumeister et al., 2001; Rozin and Royzman, 2001), are captured by the SDRST (Belham et al., 2013).

### Non-Human Primates

**Figure 4:**
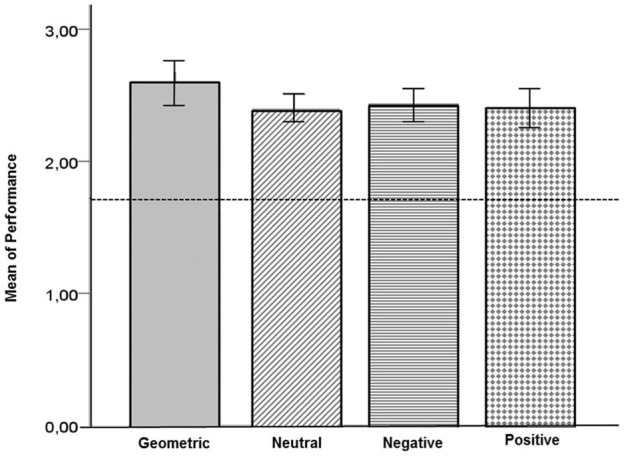
**Mean performance of adult captive Capuchin monkeys (*Sapajus libidinosus*) on the SDRST with geometrical, neutral, negative and positive stimuli**. The line represents chance level *N* = 5.

The SDRST has also been successfully used with non-humans primates (Belham et al., 2014). Five captive adults of the species *Sapajus libidinosus* (Spix 1823) aged between 11 and 16 years old answered the task in the same conditions as the human subjects but also receiving a food reward after each correct response. The animals are kept in groups of three to five in cages with access to natural environmental conditions. Their performance was above chance for geometric images and for facial photographs, indicating that they were capable of learning the task rules (Figure 4). This suggests that this computer-based SDRST can be used in comparative studies between species. This study was approved by the Ethics Committee for Animal Use of the Institute of Biological Sciences of the University of Brasilia (UnBDOC n° 63853/2011).

### Electrophysiological Data

Studies “b” and “c” also measured the cortical brain activity of participants while responding to the SDRST. Electroencephalographic (EEG) data were recorded from 21 scalp channels following the 10–20 international system, plus two reference electrodes on the mastoids. Continuum records were made with a NeuroSpectrum 4EP system (Neurosoft, Russia) with impedances kept below 5 kΩ and a 2000 Hz sampling rate. Data were then processed with EEGLAB v.9.0.4.5 (Delorme and Makeig, 2004),^1^ separated into non-overlapping epochs time-locked to each stimulus category. Eye movement and blink artifacts were removed with the Independent Component Analysis (ICA, Bell and Sejnowski, 1995). For the results, data were divided into the traditional frequency bands: theta (4–8 Hz), alpha (8–13 Hz), beta (13–30 Hz) and gamma (30–70 Hz) (Garcia et al., 2011; Belham et al., 2013).

**Figure 5:**
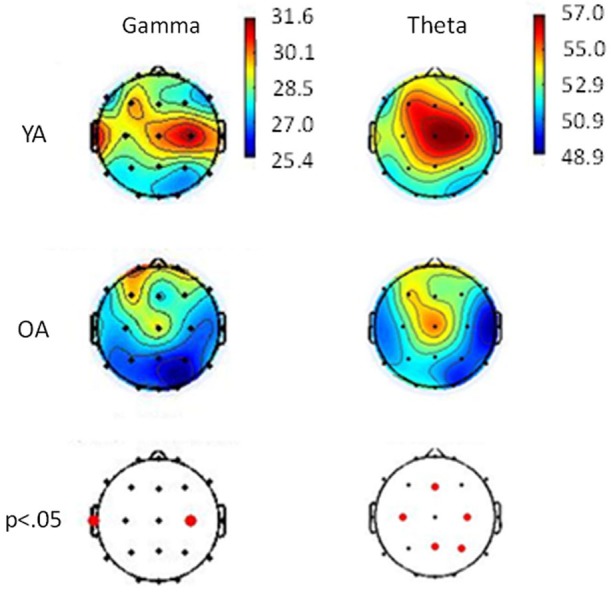
**Relative topographic power spectrum distribution for Gamma (30–70 Hz) and Theta (4–8 Hz) bands with geometrical stimuli for each young adults (YA) and older adults (OA)**. Red dots indicate significant statistical differences (*p* < 0.05; parametrical tests) related to age groups in electrode location.

Data analysis showed cortical activation compatible with what is known for WM and spatial information studies. There was a greater activation in prefrontal and frontal areas of scalp, which is consistent with the type of activity seen for WM tasks (Speck et al., 2000; Jonides et al., 2005). There was also a higher theta band activity on the midline region of the scalp, which is thought to be related to attention, concentration and mental effort (Gevins et al., 1997; Onton et al., 2005), all abilities required to a successful response to a WM task. Besides that, gamma activation in the prefrontal and left temporal regions, like the one found, is related to codification of visual stimuli (Düzel et al., 2010) and to the maintenance of spatial information in WM (Jacobs et al., 2006; Jokisch and Jensen, 2007). OA displayed a smaller activation on central areas of the scalp when compared to YA, which may be related to their lower performance (Figure 5). For the complete description of analyzes and results regarding electrophysiology data, see Belham et al. (2013) and Garcia et al. (2011).

## Conclusions

The present paper describes a new computer-based task to evaluate visuospatial WM abilities, in which participants are required to maintain the spatial information of a crescent sequence of items online and make decisions about them. We show that this version of the Spatial Delayed Recognition Memory Task is sensitive to the natural age-related cognitive differences and also to the deficits brought by AD. We also present data indicating that the manipulation of type, complexity, variability and emotional valence of stimuli is possible and successful in studies about WM load. The fact that capuchin monkeys were able to complete this task in a touch-screen monitor will allow future comparative studies between species. Electrophysiological findings corroborate that areas known to be related to executive functions, WM and decision making are activated during this task. Therefore, this new version of the SDRST seems to be a reliable and sensitive measure to investigate visuospatial WM in different groups of participants, including clinical and non-clinical populations. It may represent a useful and effective cognitive measure to detect cognitive changes associated with normal aging and dementia. From a public health point view, the new approach could help to collect large-scale data from aged people in general population in the world by connecting the system to the internet. Mobility and free cost of the application (SDRST app available to download for free in Apple Store app) are also benefits of the tablet device approach.

## Authors and Contributions

CS, FSB, AG, CT, MCHT conceptualized and designed the work. CS, FSB, AG acquihired and analysed the data. CS, FSB, AG, CT, MCHT interpreted the data. CS, FSB drafted the work. CS, FSB, AG, CT, MCHT critically revised and approved the manuscript.

## Conflict of Interest Statement

The authors declare that the research was conducted in the absence of any commercial or financial relationships that could be construed as a potential conflict of interest.

## Footnotes

^1^http://sccn.ucsd.edu/eeglab/
